# A novel intraoral neuromuscular stimulation device for treating sleep-disordered breathing

**DOI:** 10.1007/s11325-021-02355-7

**Published:** 2021-03-26

**Authors:** Bhik Kotecha, Phui Yee Wong, Henry Zhang, Amro Hassaan

**Affiliations:** Queen’s Hospital, Barking, Havering and Redbridge University Hospitals NHS Trust, Rom Valley Way, Romford, Essex, RM7 0AG UK

**Keywords:** Intraoral device, Awake neuromuscular stimulation, Primary snoring, Mild OSA

## Abstract

**Purpose:**

To ascertain the usefulness of a novel intraoral neuromuscular stimulation device in treating patients with primary snoring and mild obstructive sleep apnoea (OSA). This device uses daytime awake neuromuscular electrical stimulation (NMES) as an application to induce toning of the tongue muscles.

**Methods:**

A prospective cohort study of 70 patients with sleep-disordered breathing was conducted. Objective snoring and respiratory parameters were recorded with 2 consecutive night WatchPat sleep studies before and after treatment. The device was used for 20 min once daily for a 6-week period. Secondary outcome measures using visual analogue scale reporting of snoring by patient and Epworth Sleepiness Score (ESS) were recorded. Quality of life parameters were also noted.

**Results:**

Objective reduction of snoring was noted on the sleep studies in 95% of participants, with an average snoring time reduction of 48%. Subjectively, the visual analogue scale reported by partners’ similarly demonstrated reduction in 95% of the patients with an average reduction of 40%. In a subset of 38 patients with mild OSA, AHI reduced from 9.8 to 4.7/h (52% reduction), ODI 7.8 to 4.3/h (45% reduction), and ESS from 9.0 to 5.1. Adverse effects encountered were minimal.

**Conclusion:**

This prospective cohort study demonstrates a notable improvement in both objective and subjective parameters of snoring and mild OSA in both simple snorers and patients with mild OSA. This device offers a safe and novel approach to reduce snoring and mild OSA by utilising intraoral neuromuscular electrical stimulation. This could be a preferred option for patients as it alleviates the need of using an oral device during sleep.

**Trial registration:**

clinicaltrials.gov identifier NCT03829956

## Introduction

The spectrum of sleep-disordered breathing (SDB) encompasses disorders from primary snoring (PS) to obstructive sleep apnoea (OSA) characterised by the common pathophysiology process of repeated and recurrent collapse of the upper airway during sleep. These repeated airway obstructions are significant as they result in recurrent nocturnal asphyxia, fragmented sleep, major fluctuations in blood pressure and increased sympathetic nervous system activity [1].

Furthermore, patients with untreated SDB, especially moderate or severe OSA, are at increased risk of hypertension, stroke, heart failure, diabetes, depression, road traffic accidents and cognitive dysfunction [2–5].

Although historical data traditionally states the prevalence of 4–8% of OSA in the population, literature reflects a significant increase in the prevalence over the last few decades. Recent UK survey of more than 1200 adults, as well as polysomnographic data from a Swiss community sample of over 2000 individuals aged 40 to 85, indicates an increase in prevalence of SDB [6, 7]. Similarly, the estimated prevalence of moderate to severe OSA from the Wisconsin sleep cohort study in the USA has increased from 14 to 55% over the past two decades [8]. Recent study by Benjafield et al. has addressed the issue of global prevalence of OSA and collated data from 17 different countries with China, the USA, Brazil and India being reported as amongst the highest affected [9]. They calculated that nearly 1 billion adults aged 30 to 69 are estimated to have OSA globally, with some countries having prevalence of more than 50% [9].

It has been well established that a crowded or narrow upper airway is a common contributing factor in SDB. Various studies using imaging techniques consistently demonstrate that, on average, the static cross-sectional area of the pharyngeal airway in people with OSA is smaller when compared to their non-OSA counterparts [10]. However, as OSA does not occur during wakefulness, there must be an additional mechanism contributing to the SDB rather than just an anatomical problem, and in this respect, the reduction in airway muscle tone and alteration in the neural drive are considered to be the most important precipitating factors [11, 12]. The most notable change that occurs in the physiology of humans during sleep is the reduction in the tone of the muscles (especially the tongue) and increased collapsibility of the pharyngeal lumen. The genioglossus is considered the largest muscle of the airway and the most important dilatory muscle during sleep, and with onset of sleep, there is a rapid reduction in pharyngeal and tongue muscle contractility [13].

Treatment modalities for SDB are fairly diverse and include conservative lifestyle changes, appliances such as continuous positive airway pressure therapy (CPAP), mandibular advancement devices (MAD) and surgical intervention [14]. Surgical results are not always favourable in the long-term, and compliance with appliances remains an issue [15, 16]. Furthermore, though the recent introduction of surgical technique of hypoglossal nerve stimulation appears attractive, it is considered to be expensive and relatively invasive [17]. A recent meta-analysis study concluded that oropharyngeal exercises can reduce apnoea-hypopnoea index (AHI) by 50% [18]. The principle of training the upper airway muscles to address the airway obstruction seen in patients with OSA presents a promising and attractive alternative therapy option.

There is a considerable body of evidence to claim that the use of transcutaneous electrical stimulation in paralyzed or inactive limbs significantly improves muscle power and tone recovery [19]. Considering the muscles of the throat and tongue are of the similar skeletal muscle type as of the limbs, it seems logical that electrical stimulation of the pharyngeal and tongue muscles could lead to a similar effect of increased resting muscle tone and muscle tone during sleep.

The first proof of concept of daytime awake stimulation of the tongue was reported by Wiltfang et al. in 1999, demonstrating that when compared to placebo, daytime active stimulation of the tongue muscles for 2 weeks resulted in a significantly improved respiratory disturbance index (RDI) and nadir oxygen saturation levels [20]. In a further study, using an external daytime neck stimulator for an average of 4 weeks noted a significant drop in both AHI from 29.2 to 21.2 and in the partners witnessed snoring scale from 7.0 to 3.4 on a visual analogue scale [21].This interesting concept of using submental transcutaneous electrical stimulation in isolation or combined with a single intraoral electrode on the floor of mouth leading to objective improvement in SDB was instrumental in developing the novel device eXciteOSA® (formerly known as Snoozeal) presented in this study. The eXciteOSA® device uses an entirely intraoral appliance, resting directly on the very conductive wet surface of the tongue, with a pair of electrodes above and a pair below the tongue to ensure vertical and diagonal patterns of stimulation.

## Materials and methods

This prospective cohort study (formal ethical committee approval was attained, and the study was registered: clinicaltrials.gov identifier NCT03829956) on individuals with primary snoring or mild OSA was performed on 75 patients. Patients were recruited from Queen’s Hospital, Barking, Havering and Redbridge University Hospitals NHS Trust, Romford, Essex, and from the private practice sector in the UK. Patients had to be aged eighteen or above and been suffering from habitual snoring (snoring for more than 5 out of 7 nights per week) for more than 6 months. They were also required to have a live in partner. Patients with body mass index (BMI) of greater than 35 and AHI of greater than 15 were excluded as were patients with symptomatic nasal pathology, tonsillar hypertrophy at grade three or above and patients with tongue piercing, pacemakers or implanted electrical medical devices. Patients who had previous oral surgery for snoring and those with relevant facial skeletal abnormalities were excluded. Patients were fully informed and appropriately consented.

A full clinical history was obtained from each patient, and they all had their BMI, neck collar size and Epworth Sleepiness Score (ESS) recorded and underwent a thorough clinical examination by the senior author which included a fibre-optic flexible endoscopic evaluation of the nose, pharynx and larynx. The salient features within the clinical evaluation that were focused on included nasal framework abnormality, nasal pathology, tonsillar hypertrophy grading, Friedman tongue position grading, simulated snoring, Müllers Manoeuvre, degree of lymphoid hyperplasia of the tongue and impact of protruding the mandible forward as in the Esmarch manoeuvre.

The study assessed objective snoring (% time snoring, different loudness levels) and respiratory parameters (AHI, ODI and oxygen saturations) with two consecutive night sleep study (WatchPat 200 Unified WP200U) before being recruited in the trial and again two consecutive night sleep study after completing the study. This data was supplemented with bed partner visual analogue scale (VAS) reporting snoring intensity and sleep quality questionnaires—Epworth Sleepiness Scale (ESS) and Pittsburgh Sleep Quality Index (PSQI). The eXciteOSA® device was used for 20 min, once a day for a 6-week period. The patient and the bed partner were provided with a research booklet in order to record the pre-treatment, treatment period and two-week post-treatment period data.

The patients were given information sheet on the eXciteOSA® device and a lengthy discussion, and explanation about how to use the device was shared.

The eXciteOSA® device targets the intrinsic and extrinsic tongue muscles by delivering neuromuscular electrical stimulation to the back of the tongue with the purpose of increasing muscle tone and preventing excessive relaxation. The device has been approved for use by the European Union, Australian TGA, and Health Canada and currently awaits approval by the FDA in the USA.

**Fig. 1:**
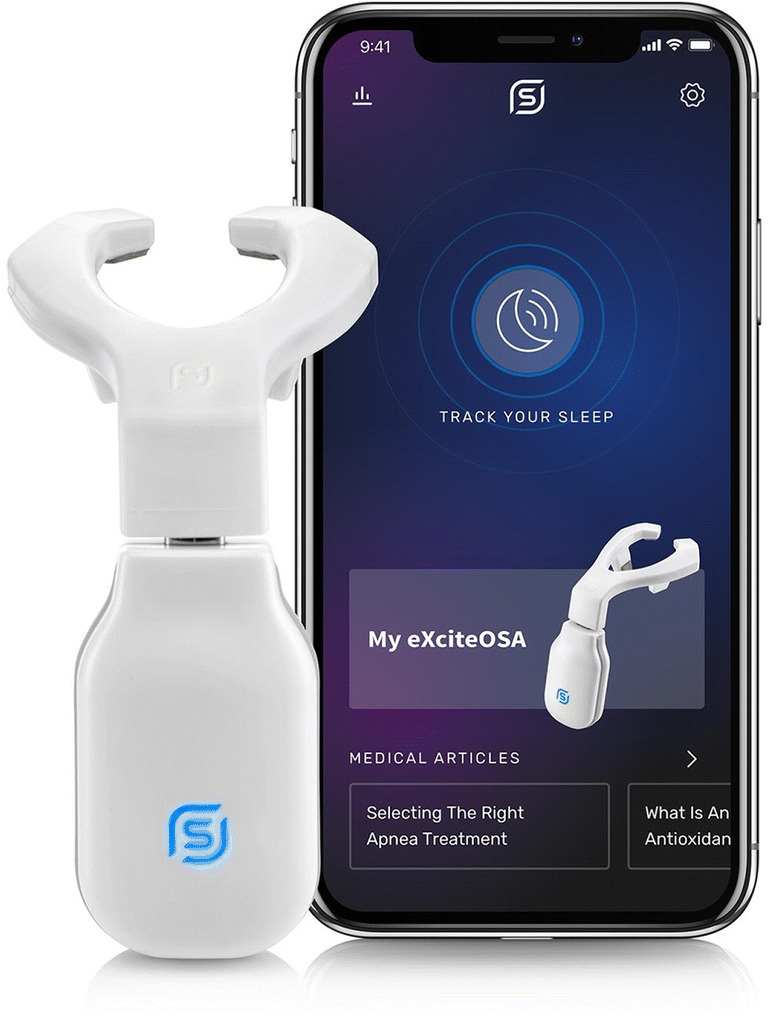
eXciteOSA device with smartphone app

The device consists of three components (Fig. 1):

1. Washable flexible mouthpiece with electrode array that fits onto the tongue.
2. Rechargeable control unit that attaches to the mouthpiece via a USB-C connection.
3. Smartphone app that manages the functions of the device.

**Fig. 2:**
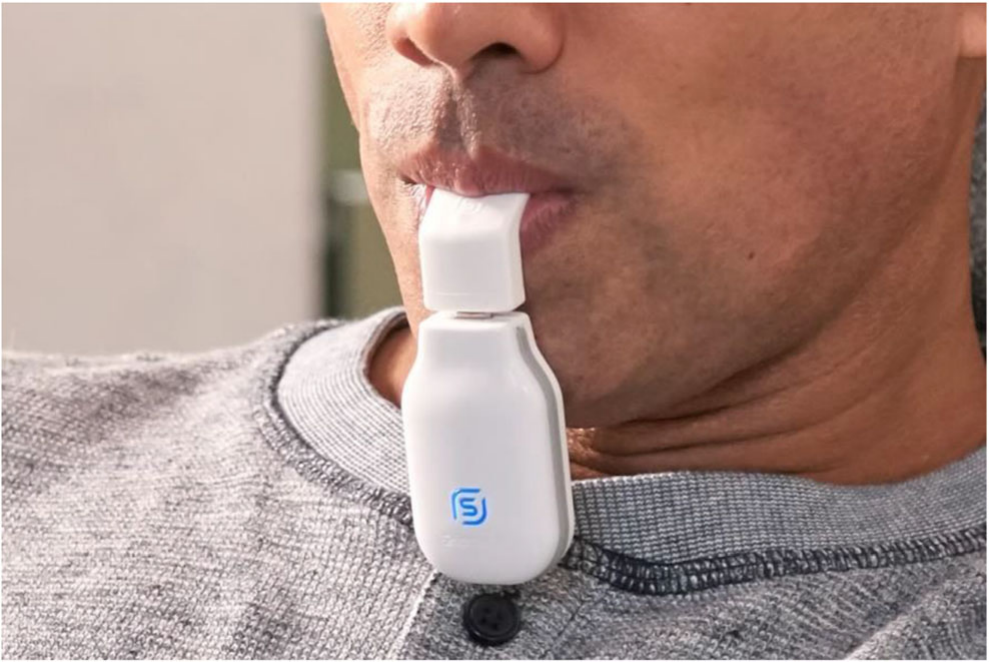
eXciteOSA device in situ

The patients were instructed to insert the mouthpiece with two electrodes located above and two electrodes located below the tongue (Fig. 2). Bipolar biphasic current was delivered with predetermined stimulation and rest periods, migrating between three low frequencies (0–20Hz). The intensity of therapy (maximum of 15mV) was controlled by the patient and advised to use the maximal tolerable intensity without discomfort. It was anticipated that such device could help patients with SDB and was indeed supported by the first proof of concept study conducted between centres based in Germany and the UK [22]. The therapy consisted of a series of pulse bursts with intensity controlled to a tolerable level by patient, for 20 min during wakeful state for a period of 6 weeks. With daily use of eXciteOSA®, the tongue muscle function was expected to improve in order to prevent it from collapsing backwards and obstructing the airway during sleep. The device was able to detect utilisation and hence the compliance via smartphone app and bluetooth technology. The patient’s partner was instructed to score the VAS 2 weeks prior to commencing the treatment and during the 6 weeks of the treatment. The quality of life and sleep questionnaires were similarly completed. All patients had two-night pre- and post-treatment sleep study.

Primary outcome measures included reduction in snoring levels at greater than 40 dB. Any objective change in terms of improvement or otherwise at this level was extracted from the WatchPat study report. Furthermore, visual analogue scale snoring score during and post-therapy was recorded as part of primary outcome measure too. Pittsburgh sleep quality index and Epworth sleepiness score were recorded as subjective secondary outcome measures. Both objective and subjective data analyses were performed using SPSS (version 23) statistical software programme. Objective parameters assessed were snoring duration and intensity, AHI, ODI and RDI as evaluated by the WatchPat sleep study apparatus. Subjective parameters studied included ESS, PSQI (for participant and bed partner), participant subjective sleep quality, bed partner snoring record visual analogue scale (BP VAS) as well as any adverse effect encountered whilst using the device. Furthermore, we tried to establish if there were any demographic factors or endoscopic examination predictors that were associated with responders. Statistical analyses were performed using paired sample *t*-test and/or independent sample *t*-test where applicable. Non-parametric counterpart tests were also performed. Logistic regression was performed by considering 4 different groups of explanatory variables, e.g. demographic variables, sleep study parameter variables, clinical examination variables and, finally, endoscopy examination parameters. The effect of each set of the explanatory variables on response was assessed by performing multiple logistic regressions with all explanatory variables included. We did not encounter the problem of overfitting. However, in a few cases, highly non-significant explanatory variables were removed manually from the analysis to have more robust standard errors. In some cases, also non-significant variables were carefully removed by a step-wise logistic regression.

## Results

Of the 75 patients recruited, 5 dropped out and 70 (44 males, 26 females) completed the trial. The average age ranged from 24 to 79 (mean 46.45) and the BMI from 20.4 to 34 (mean 26.7). Of the 5 that dropped out, one did so because of strong gag reflex and therefore was unable to tolerate the device, one lady became pregnant, one participant had very poor dental hygiene and the remaining two withdrew for personal reasons. Of the 70 patients, 32 were simple snorers, and 38 had mild OSA with AHI between 5.05 and 14.8/h. The baseline demographic data is illustrated in Table 1. Snoring data was analysed in all patients, and the respiratory parameters more specifically were studied in the mild OSA sub-group.

**Table 1:** Baseline demographic data of the treated patients

	*N*	Min	Max	Mean	Standard deviation
Age	70	24	79	46.457	14.7055
BMI	70	20.4	34	26.7857	3.35344
Pre AHI	70	0.2	14.8	5.9479	4.031709
Pre ESS	70	0	22	8.9	4.86454
Alcohol unit/week	70	0	40	5.1071	7.54834
Smoking pack years	70	0	7	1.4157	5.26576
Neck collar (cm)	68	31.75	48.26	38.6	1.52281

### Objective data

Objective measurement of snoring is a complex and poorly defined entity. There are no agreed or published guidelines on which parameters of snoring that should be measured or what degree of change can be considered clinically relevant. Comparative analysis is difficult as within the literature, different trials report on different indices. WatchPat sleep study reports on the % time the individual snores at different thresholds with a fixed position of the recording mic at the sternal notch.

The objective change in snoring time was noted at snoring intensity threshold level of 40 dB (all snoring), 45dB (moderate snoring) and 50dB (epic snoring) and demonstrated an improvement in 66 (94.8%), 62 (88.5%) and 61 (87.1%) of the 70 patients respectively. Table 2 illustrates relation to the reduction of snoring time at three different intensities and demonstrate statistical significant improvement at each level. The average reduction in snoring at each of those intensity levels and the actual reduction in snoring time at 40, 45 and 50 dB levels were 40.8%, 46.6% and 40.9% respectively. As any improvement in snoring could be considered as a liberal entity, we therefore chose to further calculate an improvement of greater than 25% in the snoring to be a more stringent perspective and noted that 74.3% of the patients in our study had demonstrated an improvement of this calibre at 40dB.

**Table 2:** One-sample test illustrating % changes in snoring at three different sound intensity levels

	% Time snoring Pre-therapy	% Time snoring Post-therapy	Mean % reduction	Sig. (2-tailed) *P* value	95% confidence interval of the difference	
					Lower	Upper
Change in snoring greater than 40 dB	29.05	16.76	40.84	<0.001	34.3105	47.3785
Change in snoring greater than 45dB	12.52	5.75	46.64	<0.001	37.5997	55.6739
Change in snoring greater than 50dB	6.64	2.92	40.94	<0.001	30.6553	51.2310

Multivariate analysis was performed to identify any predictors of success for snoring improvement at 40dB threshold. Multiple logistic regressions were the method used to determine the predictors for improvement in snoring. AHI was included in the multivariate analysis, and there was no association found. Pre-treatment AHI was used as a continuous variable in the logistic regression analysis of sleep study parameters. However, to assess the effect of three different categories of AHI (<5, 5–10, >10), it was therefore also included as a categorical variable and remained non-significant.

There was a positive association with ESS (i.e. the higher chance of response, the higher the pre-therapy ESS with odds ratio of 1.194) and negative correlation with BMI (odds ratio of 0.0805). There was no association with demographic indices (age, sex, average alcohol intake, smoking), with clinical indices (neck collar size, Nasal pathology, tonsil size, Friedman tongue position) or endoscopic evaluation (endoscopic characteristics, Muller Manoeuvre-related collapse, simulated snoring or Esmarch manoeuvre). There was no obvious significant association between intensity levels of stimulation and reduction in snoring. However, this was not an objective for this particular study.

**Fig. 3:**
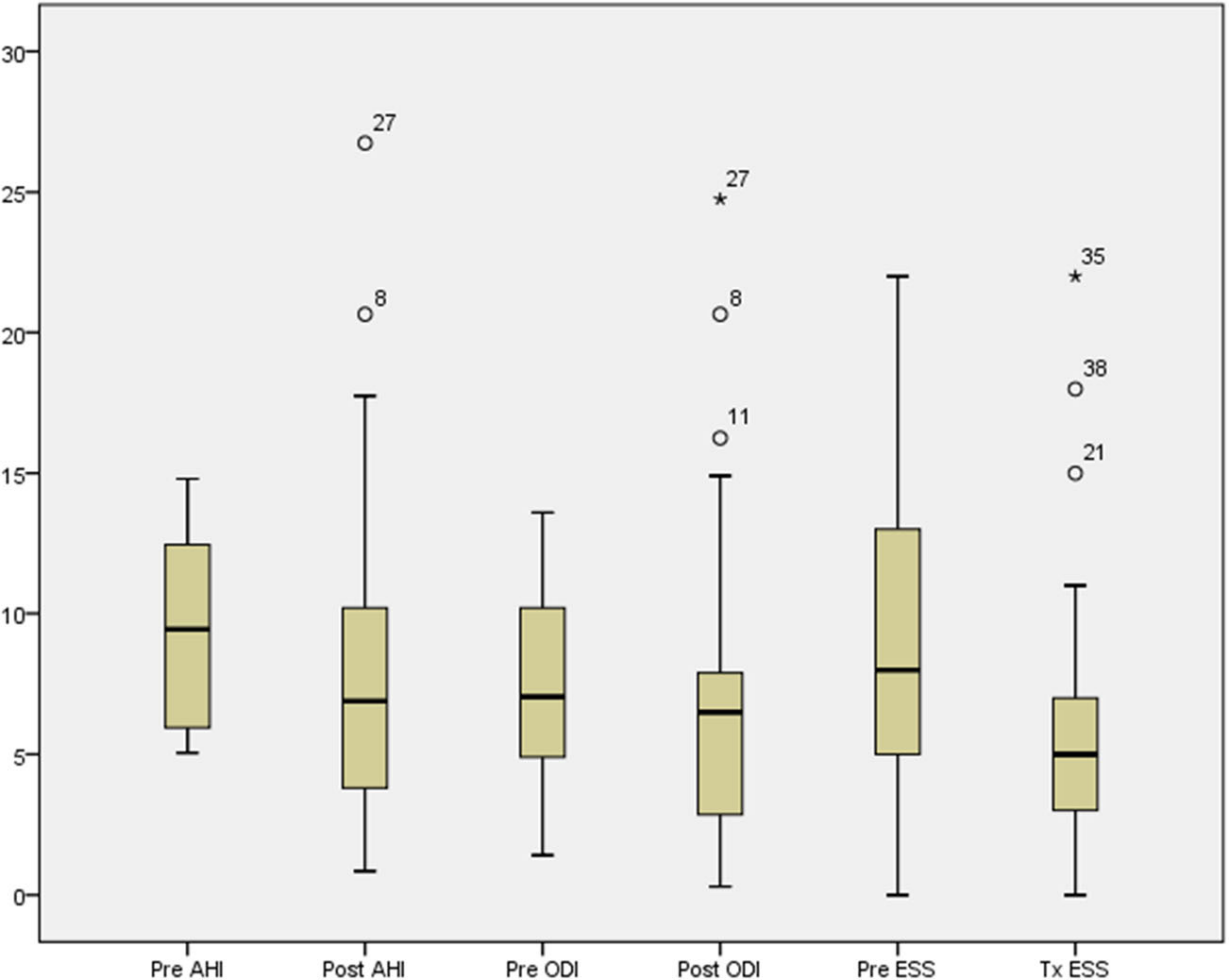
Change in AHI, ODI and ESS in subgroup of patients with mild OSA (*n*=38) (*p*<0.001)

The AHI in this cohort of 70 patients ranged from 0.2–14.8/h, and the mean AHI value was found to be 5.94/h which dropped to 5.37/h following completion of eXciteOSA® therapy. Likewise, the mean ODI reduced from 4.92 pre-treatment to 4.73 post-treatment. However, in patients with mild OSA (*n*=38), the mean AHI dropped from 9.8 to 4.7/h and the ODI from 7.8 to 4.3/h (Fig. 3). These changes were statistically significant (*p*<0.001). It was noted that the greatest reduction of AHI was in the patients with the initial value of AHI greater than 10/h. We evaluated the impact of supine sleep position on our outcomes and noted that the amount of time spent in supine position was 46% and 42% pre- and post-treatment respectively, and this was not statistically significant.

There was a corresponding significant reduction in ESS from 9.0 to 5.1 (*p*<0.001) for this subgroup of 38 patients (Fig. 3).

**Fig. 4:**
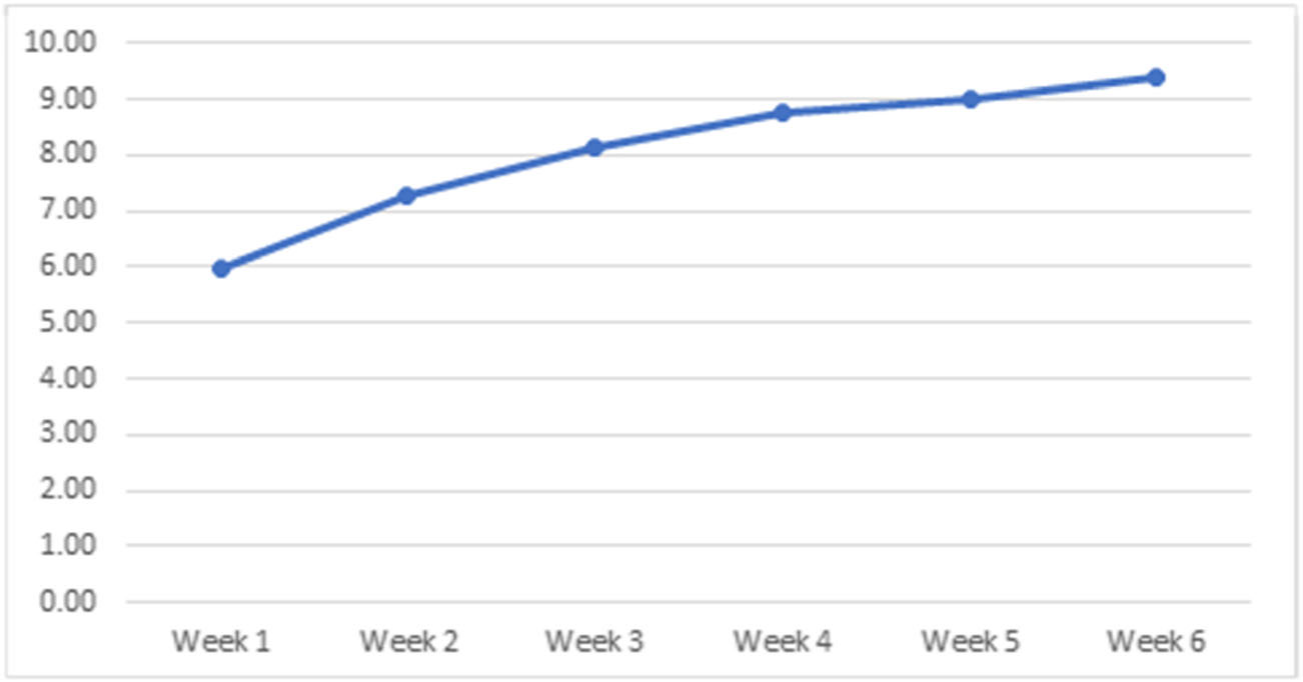
Device stimulus intensity levels during the 6-week treatment period

When evaluating stimulus intensity (level range 1–15) utilised by participants, we found that the average intensity starting point was at level 6 (equivalent of 18mA), and by week 6, the average tolerable intensity had increased to 9.3 (Fig. 4). Device compliance was measured remotely by app utilisation, and this demonstrated a range of 59.5–95.2% with the average value of device utilisation being 83.3%.

### Subjective data

**Fig. 5:**
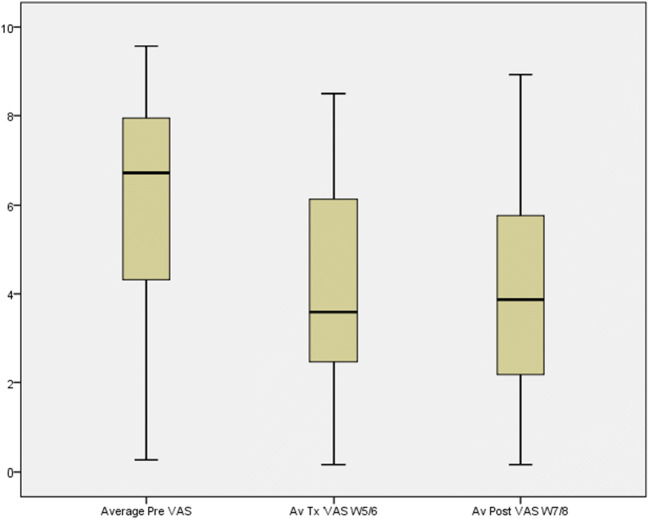
Bed partner visual analogue score (VAS) before treatment, end of treatment (week 5/6) and after stopping treatment (week 7/8) (*p*< 0.001)

Of the 70 patients, 61 completed the snoring VAS forms adequately. The pre-treatment mean snoring VAS dropped significantly (*p*<0.001) from 5.88 to 3.98 at week 5/6 of the treatment period, and this benefit was sustained at week 7/8 despite stopping the treatment (Fig. 5).

All 70 patients had completed the ESS form pre- and post-treatment, and the mean respective values were 8.4 and 6.14 and which was statistically significant (*p* < 0.001). Total PSQI (patient) and total bed partner PSQI decreased significantly (Table 3). With regard the PSQI, the most significant decrease was seen with components 1 (subjective sleep quality) and 5 (Sleep disturbance).

**Table 3:** Sleep quality parameters of participants and partner

	Mean	*P* value	Standard deviation
Pre-therapy ESS Post-therapy ESS	8.9815	<0.001	4.86454
	6.4630		3.88712
Bed partner pre ESS Post-therapy bed partner ESS	5.7708	0.205	4.10324
	5.0000		3.74122
Pre-therapy total PSQI Post-therapy total PSQI	7.0294	0.004	3.12979
	5.9216		2.83246
Bed partner pre-therapy PSQI Bed partner post-therapy PSQI	7.3488	0.029	2.76128
	6.3256		2.79651

In terms of adverse effects secondary to the use of eXciteOSA® device, 11 participants had commented on experiencing mild problems at some stage during the 6-week therapy period. This included excess salivation by 10 (14.2%) patients, tongue tingling/discomfort by 7 (10%) patients, filling sensitivity by 3 (4.2%) patients, metallic taste and gagging sensation by 3 (4.29%) patients each and tightness in the jaw by 1 (1.43%) patient. In all cases, symptoms were very mild and only lasted during or part of the 20-min therapy time. There were no long-term problems reported.

## Discussion

The strategy of one size fits all is not appropriate for a multifactorial diverse condition such as sleep-disordered breathing. Furthermore, although conventional therapies alleviate the obstruction when in use, they fail to modify the disease and can suffer from low compliance. Daytime neuromuscular electrical stimulation (NMES) treatment for correction of night-time airway obstruction is a novel, innovative and probably unconventional therapeutic strategy. However, the possibility of reversing the pathophysiology of SDB and not having a night-time wearable makes this an attractive strategy to explore. This is particularly useful when comparing this device to the MAD where the patient has to retain this throughout the night and can have a notable adverse effect on dental occlusion and may cause TMJ dysfunction and dental movement.

NMES involves the application of an electric current through electrodes placed over targeted muscles, to induce muscular contractions and has been shown to activate the muscle to a greater extent than voluntary muscle actions under identical conditions [19]. It has also been used to induce the activity of motor units that are difficult to activate voluntarily [19]. NMES has been shown to result in a change in myofibrillar protein expression to induce a phenotype shift of fatigue-prone to fatigue-resistant (i.e. fibre type IIb to I or IIa changes) with strengthening of the cytoskeleton [23]. NMES has also been shown to result in muscle metabolic shift from glycolytic to oxidative profile, increased intracellular defence against harmful oxygen species, reverse the degenerative pre- and postsynaptic tongue neural morphology associated with ageing and a shift of small to large diameter muscle fibre size with higher contractile tensions [23, 24].

eXciteOSA® device provides a targeted retraining tool to stimulate the biggest dilatory upper airway muscle (tongue and genioglossus) and, furthermore, has the added advantage of not interfering with the sleep as can be the case of other devices utilising continuous transcutaneous electrical stimulation in the submental region [25, 26]. This study confirms its usefulness in improving mild sleep apnoea, snoring and sleep quality.

Objective measurement of snoring is a complex and poorly defined entity. There are no agreed or published guidelines on which parameters of snoring that should be measured or what degree of change can be considered clinically relevant. Comparative analysis is difficult as within the literature different trials report on different indices. WatchPat sleep study reports on the % time the individual snores at different thresholds with a fixed position of the recording mic at the sternal notch. This device demonstrated a 95% response rate in some objective reduction in snoring (all snoring above 40 dB) with an average reduction of 41% for the whole group. Furthermore, the degree of change noted increases as the threshold of snoring is increased, suggesting that higher decibel snoring has a greater reduction.

The changes in snoring sound are supported by a reduction in airway obstructive events. In the subset of mild OSA patients (*n*=38), a 52% reduction in AHI and 45% in ODI were noted. The reduction in snoring, AHI and ODI all concur with a reduction in airway resistance. For a 20-min once a day therapy with no night time wearable, this offers a clinically significant and effective change in objective snoring and mild OSA.

The reduction in snoring time and obstructive events was associated with improvement in sleep quality, PQSI and ESS. The bed partner PQSI also improved. This study differed slightly from the original proof of concept study by Wessolleck et al. [22] in that the latter utilised the device twice for 20 min each whereas our study recommended it to be used just once. This was to minimise difficulties in compliance associated with twice daily use. In spite of this, the results attained in this study are similar and comparable to the Wessolleck paper.

One primary limitation of this study is lack of controls. Furthermore, considering the exclusion criteria in the study, efficacy in patients who may have had previous oropharyngeal surgery has not been established. Adverse effects encountered in this study secondary to the eXciteOSA® device were very minimal and short lived only troubling the patient for short duration of 20 min with no long-term or permanent effects. This in addition to the fact patient has full control on the device via the remote control or the smartphone app makes the device inherently safe and attractive for utilisation in treating SDB. All participants found this device easy to use. It would be interesting to establish the sustenance of the improvement noted at 2 weeks post-therapy, and we have further studies planned to include 3- and 6-month follow-up evaluation. In addition, we are hoping to assess the impact of either periodic or continual stimulation impact on expected treatment regimen and long-term outcomes. For this particular study, we have not conducted specific temporal analysis to ascertain exactly when the improvement in symptoms is first noted, and this is also a proposed future research project.

## Conclusion

Our understanding of the mechanisms of SDB is evolving. Although a narrowed upper airway is a common identifiable characteristic, increasing understanding of the neural control, airway muscle responsiveness/effectiveness and central response to increased intrathoracic pressures is changing our paradigm and management strategies for SDB and OSA. The future is likely to be more bespoke therapy(s) and move away from one size fits all. To achieve this target, we need reliable methods of assessing our patients and a larger variety of therapies that target these physiological deficiencies. We believe that eXciteOSA® device offers a novel approach with a minimally invasive treatment modality for SDB, and this study provides both objective and subjective supportive data in terms of snoring reduction and mild OSA. Furthermore, the improvement in sleep quality reflected in post-treatment values of PSQI and ESS is indeed very encouraging.
